# Plasma Amino Acids in Horses Suffering from Pituitary Pars Intermedia Dysfunction

**DOI:** 10.3390/ani12233315

**Published:** 2022-11-27

**Authors:** Sabita Diana Stoeckle, Detlef Timmermann, Roswitha Merle, Heidrun Gehlen

**Affiliations:** 1 Equine Clinic: Surgery and Radiology, Freie Universitaet Berlin, 14163 Berlin, Germany; 2 MembraPure GmbH, 16761 Hennigsdorf, Germany; 3 Institute for Veterinary Epidemiology and Biostatistics, Freie Universitaet Berlin, 14163 Berlin, Germany

**Keywords:** plasma amino acids, PPID, endocrine disease, arginine, asparagine, citrulline, cysteine, glutamine

## Abstract

**Simple Summary:**

Pituitary pars intermedia dysfunction (PPID), also known as equine’s cushing syndrome, is one of the most common diseases of aged horses and ponies. The pathogenesis of PPID includes oxidative damage to dopaminergic pathways, similar to Parkinson’s disease in humans. Here, alterations in the concentrations of the serum amino acids were reported previously. To examine changes in the plasma amino acid profile in horses with PPID, EDTA plasma of horses that were presented for various reasons that required laboratory examinations of blood anticoagulated with EDTA was collected. With this plasma, the basal ACTH concentration, as well as the amino acid profile, was determined. The basal ACTH concentration is commonly used to diagnose PPID. Horses were considered PPID patients if the ACTH concentration was ≥ 100 pg/mL, i.e., they would be considered affected at any time. Horses were defined as non-PPID (nPPID) patients if the ACTH concentration was below 30 pg/mL. PPID is commonly treated with pergolide. Horses receiving pergolide with ACTH ≤ 30 pg/mL were allocated to the group PPIDrr (PPID, ACTH in reference range) and horses receiving pergolide with ACTH ≥ 100 pg/mL to the group PPIDarr (PPID, ACTH above reference range). In total, 93 horses were examined, including 88 horses at the clinic and 5 horses at a private practice. Of these, 53 horses fulfilled the inclusion criteria (ACTH ≤ 30 pg/mL or ACTH ≥ 100 pg/mL). A total of 25 horses were diagnosed as nPPID, 20 as PPID, 5 as PPIDrr, and 3 as PPIDarr. Arginine was significantly higher in PPIDrr than in PPID and nPPID, asparagine was significantly higher in PPID, PPIDrr, and PPIDarr than in nPPID, citrulline was significantly higher in PPIDrr than in nPPID and PPID, cysteine was significantly lower in PPIDrr than in PPID, nPPID, and PPIDarr, and glutamine was significantly higher in PPID and PPIDarr than in nPPID. Especially, asparagine, citrulline, and glutamine may be potential diagnostic markers and may offer interesting approaches for research regarding amino supplementation in PPID.

**Abstract:**

Pituitary pars intermedia dysfunction is one of the most common diseases of aged horses and ponies. In Parkinson’s disease, which is, similar to PPID, a disease that involves oxidative damage to dopaminergic pathways but with different clinical signs, alterations to the serum amino acid profile have been reported. To examine changes in the plasma amino acid profile in horses with PPID, EDTA plasma of horses that were presented for various reasons that required laboratory examinations of blood anticoagulated with EDTA was collected. With this plasma, the basal ACTH concentration as well as the amino acid profile was determined. Horses were considered PPID patients if the ACTH concentration was ≥ 100 pg/mL, i.e., they would be considered affected at any time. Horses were defined as non-PPID (nPPID) patients if the ACTH concentration was below 30 pg/mL. Horses receiving pergolide with ACTH ≤ 30 pg/mL were allocated to the group PPIDrr (PPID, ACTH in reference range) and horses receiving pergolide with ACTH ≥ 100 pg/mL to the group PPIDarr (PPID, ACTH above reference range). In total, 93 horses were examined, including 88 horses at the clinic and 5 horses at a private practice. Of these, 53 horses fulfilled the inclusion criteria (ACTH ≤ 30 pg/mL or ACTH ≥ 100 pg/mL). A total of 25 horses were diagnosed as nPPID, 20 as PPID, 5 as PPIDrr, and 3 as PPIDarr. Arginine was significantly higher in PPIDrr than in PPID and nPPID, asparagine was significantly higher in PPID, PPIDrr, and PPIDarr than in nPPID, citrulline was significantly higher in PPIDrr than in nPPID and PPID, cysteine was significantly lower in PPIDrr than in PPID, nPPID, and PPIDarr, and glutamine was significantly higher in PPID and PPIDarr than in nPPID. Especially, asparagine, citrulline, and glutamine may be potential diagnostic markers and may offer interesting approaches for research regarding amino supplementation in PPID.

## 1. Introduction

Pituitary pars intermedia dysfunction is one of the most common diseases of aged horses and ponies (≥15 years) [1,2,3]. Laminitis occurs in 30–40% of PPID patients and may necessitate euthanasia [4,5,6]. In PPID, hypertrophy, hyperplasia, and microadenoma or macroadenoma formation of the pars intermedia of the pituitary occurs [7]. This leads to an increased secretion of the pars intermedia-derived POMC (Pro-Opiomelanocortins) into the circulation [7]. From the healthy equine pars intermedia, only a small amount of the adrenocorticotropic hormone (ACTH) is released and it is further cleaved into α-melanocyte stimulating hormone (α-MSH), β-endorphine (β-END), and corticotropine-like intermediate lobe peptide (CLIP) [7]. In the diseased equid, the pars intermedia secretes an increased amount of POMC-derivates into the systemic circulation. An increase up to 40-fold was reported previously [8]. Nowadays, the basal ACTH concentration is a commonly used test to diagnose PPID with a sensitivity of approximately 70–80% and a spe-cificity of approximately 80–90% [5,9,10]. ACTH stimulates the adrenal gland to synthesize and release cortisol into circulation [11]. Previous research has suggested that the increased endogenous glucocorticoid concentrations may be responsible for laminitis, since these are known to be associated with systemic insulin resistance [12,13,14,15,16,17]. The suspected mechanism is that binding of insulin to the insulin-like growth factor 1 receptor (IGF-1R) has an effect in the lamellar tissue of the hoof [18,19,20] and also that insulin can directly trigger laminitis even through mechanisms that have not yet been identified [21]. In addition to its effects on the glucose metabolism, insulin is an important regulator of protein metabolism. Due to an increased utilization of amino acids, hyperinsulinemia can contribute to an increased amino acid and protein turnover, for example, in the skin or skeletal muscles [22,23,24,25,26].

Furthermore, in human Parkinson’s disease, which is, similarly to PPID, a disease that involves oxidative damage to dopaminergic pathways but has a different clinical presentation [27], the arginine, alanine, and phenylalanine concentrations were significantly lower in patients with advanced disease and dyskinesia than in patients suffering from early disease and could therefore serve as a biochemical marker of disease progression [28]. As possible reasons for the significant differences in the serum amino acid profile malabsorption and changes in amino acid metabolism, the effects of mitochondrial dysfunction and oxidative stress, reflection of progressive neurodegenerative processes in the brain, and the effect of dopaminergic medications and aromatic L-amino decarboxylase inhibitors were suggested [28].

Beside Parkinson’s disease, a change in the amino acid level in plasma or serum has been demonstrated in other human diseases, including autism and cancer. A study by Naushad et al. reported increased concentrations of glutamic acid and asparagine, and lower concentrations of phenylalanine, tryptophan, methionine, and histidine in autistic children [29]. Another study reported significantly higher concentrations of histidine, 1-methyl-histidine and 3-methyl-histidine, and significantly lower concentrations of homocysteine, carnosine, methionine, cystathionine, cystine, tyrosine, and threonine in autistic children compared to healthy children [30]. Therefore, it appears that increased concentrations of excitatory amino acids (glutamate and asparagine) and decreased concentrations of essential amino acids (phenylalanine, tryptophan, and methionine), as well as decreased concentrations of neurotransmitter precursors (tyrosine and tryptophan), may be distinctive features of the plasma amino acid profile of autistic children and that this could offer an opportunity for early diagnosis [29].

Even in disease caused by the novel coronavirus (SARS-CoV-2), significant differences in the amino acid profile in the plasma in hospitalized adults and children with multisystem inflammatory syndrome, in particular a reduced arginine concentration and arginine bioavailability, are considered important. It was suggested that arginine deficiency may contribute to endothelial dysfunction, T cell dysregulation, and coagulopathy [31]. Changes in plasma amino acid profile were also found in cancer patients. This could be due to changes due to cancer-induced protein metabolism in tumors, skeletal muscle, and liver in cancer patients. Cancer-related plasma amino acid profiles are particularly present in cancers which affect the digestive organs. These are not only influenced by the type of cancer, but also by the cancer stage [32,33,34,35,36,37]. The plasma amino acid profile seems to undergo specific changes during different diseases, not only in humans, but also in animals, which was previously shown in horses suffering from equine metabolic syndrome [38,39] or in experimentally-induced hyperinsulinemia [21]. 

Therefore, we hypothesized that there may be differences in the amino acid profile of healthy horses and horses affected with PPID. Furthermore, we assumed that treatment with pergolide may affect the amino acid profile as well and that these may be used as a potential diagnostic biochemical marker. 

## 2. Material and Methods 

### 2.1. Study Population

Included in the study were horses that were presented for various reasons at the Equine Clinic of Freie Universität Berlin (FU Berlin) that required laboratory examinations of blood anticoagulated with EDTA. The horses had to be clinically healthy except for possible orthopaedic or ophtalmological reasons for examination and/or surgery. After a physical examination, the horses were stabled. The time between transport and sample collection varied; however, all horses were given time to accommodate to their surroundings before sample collection (at least 30 min after transport). At the moment of sample collection, all horses showed no signs of pain. Their vital parameters were within normal limits and the horses were relaxed and comfortable in their surroundings. Besides the ACTH concentration, age, weight, breed, gender, and feeding regimen were recorded. Further, 5 horses that presented to a private practice to evaluate the response to therapy by determining the ACTH concentration were included in the study. Individuals with suspected or diagnosed endocrinopathies (previous dynamic testing for equine metabolic syndrome, suspicious fat accumulations) other than PPID were excluded from the statistical analysis. Since the samples were collected at different times of the year, horses were classified as healthy if the measured ACTH concentration was normal (ACTH ≤ 30 pg/mL) at any time of the year. The reference value of LABOklin (Holding—GmbH, Bad Kissingen/Germany) were used. Horses were considered PPID patients if an ACTH concentration ≥ 100 pg/mL was present, i.e., they would be considered affected at any time. Horses were defined as non-PPID (nPPID) patients if the ACTH concentration was below 30 pg/mL and as PPID patients (PPID) if the ACTH concentration above 100 pg/mL. Horses receiving pergolide with ACTH ≤ 30 pg/mL were allocated to the group PPIDrr (PPID, ACTH in reference range) and horses receiving pergolide with ACTH ≥ 100 pg/mL to the group PPIDarr (PPID, ACTH above reference range). 

### 2.2. Laboratory Diagnostics

After required laboratory diagnostics were performed (within 10 min of collection), the blood samples, uncoagulated with EDTA, were centrifuged and the plasma was separated from the solid blood components. 

#### 2.2.1. ACTH 

The plasma for the ACTH determination was sent cooled to the laboratory within a maximum of 12 h after collection. ACTH was determined with a chemoluminiscence-assay by Laboklin (LABOklin Holding—GmbH Bad Kissingen/Germany). 

#### 2.2.2. Amino Acid Concentrations 

The amino acid profiles were determined by MembraPure GmbH (Hennigsdorf/Germany). In order to determine the total cysteine concentration, bound cysteine had to be reduced to free cysteine. For this, 500 μL plasma and 100 μL dithiothreitol solution (4%) were mixed in an Eppendorf tube and incubated at 40 °C for 30 min. Then, 150 µL sulfosalicylic acid solution (10%) was added and the sample was stored for 30 min at 5–8 °C. Afterwards, 500 μL sample dilution buffer (with internal standard norleucine 100 nmol/mL) was added. The sample was centrifuged at 13,150× *g* for 5 min. After these steps, the amino acid analyses were performed with the Aracus amino acid analyzer, using the amino acid analysis method based on ion exchange chromatography with post column derivatization with Ninhydrin. The amino acid concentrations were determined by comparing the sample with a standard solution with predefined concentrations using the Clarity Chromatography Software (DataApex Company, Prague, Czech Republic).

### 2.3. Statistical Analysis

Data were analyzed using IBM SPSS Statistics version 27 (IBM Corp., Armonk, New York, USA). Data were tested for normal distribution with the Shapiro Wilk Test. The Chi-Squared-test was used to compare gender distribution between groups. Further statistical tests used were the ANOVA for normally distributed data and the Kruskal–Wallis test for non-normally distributed data. For post hoc testing, the Tukey test and the Games–Howell test or Bonferroni test were used. Laboratory data that could not be measured as they exceeded the maximum which is validated for the test or fell below the detection limit, were regarded as either the maximum (ACTH > 1250 pg/mL, n = 1) or the minimum (Asp < 5 nmol/mL, n = 20, nPPID = 7, PPID = 12, PPIDarr = 1). As per usual, the significance level was set at 0.05.

### 2.4. Ethical Statement

The study was not declared according to the German Animal Welfare law §8.1, since all samples were taken as a part of a routine clinical examination. Written owner’s consent to involve their horses in the study was obtained during the admission process at the clinic as well as at the private practice. 

## 3. Results

### 3.1. Study Population

In total, 93 horses were examined, including 88 horses at the clinic and 5 horses at the private practice. Of these, 53 horses fulfilled the inclusion criteria (ACTH ≤ 30 pg/mL or ACTH ≥ 100 pg/mL): 

A total of 25 horses were diagnosed as nPPID, 20 as PPID, 5 as PPIDrr, and 3 as PPIDarr. 

There was no significant difference regarding gender between these groups (*p* = 0.428, Welch Test). Information on breed was unavailable for two horses. Ten of the twenty individuals in the PPID group were ponies, compared to 7/25 horses in the nPPID, 1/5 in the PPIDrr, and 2/3 in the PPIDarr group. Six horses had a history of laminitis (3 nPPID, 2 PPID, and 1 PPIDarr), and there was no significant difference between the groups (*p* = 0.553, Kruskal–Wallis Test). However, significant differences between the groups were detected for feeding, age, and ACTH concentration. All horses identified as PPIDrr were exclusively maintained on a hay diet, whereas the nPPID and PPID patients also received concentrates (1 nPPID), Mash (10 nPPID, and 3 PPID) or grass (1nPPID and 12 PPID) in addition to hay (*p* = 0.005, Kruskal–Wallis Test). One horse (nPPID) received grass only. Information on age was unavailable for one horse (PPIDrr). Horses suffering from PPID were significantly older than nPPID horses (*p* < 0.001, ANOVA with Games-Howell test). Between the other groups, there were no significant differences regarding age. There were no significant differences in the ACTH concentration between nPPID and PPIDrr (*p* = 0.972, ANOVA with Games-Howell test). The ACTH concentration of PPID patients was significantly higher than in nPPID (*p* < 0.001, ANOVA with Games-Howell test), PPIDrr (*p* < 0.001, ANOVA with Games-Howell test), and PPIDarr (*p* = 0.014, ANOVA with Games-Howell test). Furthermore, PPIDarr horses had significantly higher ACTH concentrations than PPIDrr horses (*p* = 0.017, ANOVA with Games-Howell test). Mean and standard deviations of age and ACTH are displayed in Table 1.

### 3.2. Amino Acid Analysis

The mean ± standard deviation and the median, minimum and maximum of the measured amino acid concentrations are displayed in Table 2 and Table 3. The *p*-values of ANOVA or Kruskal–Wallis tests are included in these tables as well. 

Significant group differences were detected for arginine, asparagine, citrulline, cysteine, glutamine, and threonine.

Arginine in PPIDarr was significantly higher than in nPPID (*p* = 0.016, Tukey test). Asparagine was significantly higher in PPID when compared to nPPID (*p* < 0.001, Games-Howell test) and PPIDrr (*p* = 0.043, Games-Howell Test). Furthermore, the asparagine concentration was significantly higher in PPIDarr when compared to nPPID (both: *p* = 0.039, Games-Howell test). Citrulline was significantly higher in PPIDrr when compared to nPPID (*p* = 0.024, Tukey test) and PPID (*p* = 0.012, Tukey test). The cysteine concentration in PPIDrr was significantly lower than in all other groups (nPPID vs PPIDrr *p* = 0.003, Games-Howell test, PPID vs PPIDrr *p* = 0.043, Games-Howell test, PPIDrr vs PPIDarr *p* = 0.044, Games-Howell test). When compared to nPPID, the glutamine concentration in PPID (*p* < 0.001, Tukey test) and PPIDarr (*p* = 0.014, Tukey test) was significantly higher. For threonine, no significant group differences were identified by post hoc testing.

## 4. Discussion

The main limitation of this study is the insufficient number of PPIDrr and PPIDarr that were included in the analysis. However, since an adequate number of horses was included PPID and nPPID, the comparisons between these groups should provide valid results. Furthermore, even if only a few horses medicated with pergolide were available for the study, the detected differences clearly show that the amino acid profile is potentially affected by this medication. For PPID, epidemiological differences regarding gender or breed were not reported previously; however, increasing age was identified as a risk factor for PPID [1,40,41], which may explain that horses in the PPID group were significantly older than in the nPPID group. A frequently reported sign of ageing horses, loss of muscle tone, was reported by owners of geriatric horses [42,43], which was previously reported as a sign of ageing in horses [44]. Among hypertrichosis and/or other haircoat abnormalities, laminitis, lethargy, depression and weight loss, and epaxial muscle wastage or muscle atrophy are counted to be the most common clinical signs reported in horses suffering from PPID [45,46]. These changes may also be responsible for some of the changes in the amino acid profile.

In human ACTH—secreting pituitary adenoma, changes in the amino acid metabolism have been reported; particularly, these concern the alanine, aspartate, and glutamate metabolism [47]. Significant differences in the plasma concentration of asparagine, the neutral derivative of aspartic acid, were also detected in horses suffering from PPID. Healthy horses and PPIDrr patients had significantly lower asparagine concentrations when compared to PPID patients. Furthermore, PPIDarr had significantly higher asparagine concentrations than healthy horses. Additionally, higher asparagine concentrations in horses suffering from PPID compared to healthy horses have already been reported previously [48]. However, the results for asparagine must be interpreted with care since 20 horses fell below the detection limit. Also, glutamine, which was significantly lower in plasma in healthy horses than in untreated PPID patients and PPID patients who received pergolide but whose ACTH concentration was above the reference range, is synthesized from glutamic acid and ammonia [49]. An altered activity of this metabolic pathway also seems conceivable in horses suffering from PPID, especially since epaxial muscle wasting is a typical sign of PPID [45,46] and glutamine is assumed to potentially be a direct regulator of muscle synthesis and degradation [50,51]. Therefore, it could be suggested that a higher glutamine concentration in PPID patients may be assumed, since stress leads to a release of high concentrations of glutamine [52,53]. However, the storages eventually become depleted [52,53], which may explain the lower glutamine concentrations in the PPID and PPIDarr groups.

Furthermore, decreased glutamic acid, arginine, cysteine, and glutamine levels were shown to be associated with oxidative stress and neurodegeneration in Parkinson’s disease [28]. Since PPID is a neurodegenerative disease as well, decreased concentrations of these amino acids may also reflect the progression of the disease and might be used as potential markers of disease severity in the future.

The non-proteogenic amino acid citrulline was significantly higher in PPIDrr horses than in nPPID and PPID horses. This amino acid is mostly metabolized by the small intestine; therefore, it is considered to be a biomarker for the functional small intestinal bowel mass [54,55]. Furthermore, since citrulline is an intermediate metabolite in ureagenesis [56,57], during which citrulline is metabolized to arginine, a major regulator of vascular tone [58,59,60] in the kidney [61], it also is a functional biomarker for kidney function. In some cells, citrulline can act as a precursor for arginine [62] and, therefore, may be of importance for the metabolism and regulation of nitric oxide (NO) [63]. Regarding decreased citrulline concentrations and increased arginine concentrations, an upregulation of this pathway in the PPIDrr group seems conceivable; however, it should be clearly underlined again that only a few of these animals were included in the study. However, a previous study on gastrointestinally-diseased horses developing laminitis found significantly lower citrulline concentrations in horses developing laminitis than in those that did not [64]. Horses suffering from PPID are at risk for developing (endocrinopathic) laminitis [65]; however, endocrinopathic laminitis has a different pathogenesis than laminitis caused by gastrointestinal disease. Whether or not a decreased citrulline concentration is also a characteristic in equine endocrinopathic laminitis remains to be elucidated.

Cysteine is one of the least abundant, but functionally important, amino acids in proteins [66]. Mutations including cysteine residues include genetic diseases [67]. Cysteine molecules can react with other cysteine molecules by forming the typical disulfide bond, which can functionally interchange with another amino acid, selenocysteine, which is assumed to occur exclusively at functional sites [68]. A study performed on anterior pituitary cells in primary culture showed that cysteine proteases, in addition to aspartyl proteases, may be involved in the cellular metabolism of ACTH [69]. An association of these findings with lower cysteine concentrations due to altered ACTH production and pergolide treatment in PPIDrr patients remains to be elucidated, especially since these experiments were not conducted in horses but in cellular culture. Studies on human subjects with ACTH-secreting pituitary tumors also revealed metabolic changes: one study reported on 12 initially significantly changed metabolites in pituitary adenoma samples when compared to the control samples; after performing a Bonferroni correction, there were only three metabolites significantly changed: pyridoxate, deoxycholic acid, and 3-methyladipate [47]. Several changes were also detected in the metabolic pathway analysis, including changes in amino acid metabolism. Alanine, aspartate and glutamate metabolism, and starch and sucrose metabolism, as well as amino sugar and nucleotide sugar metabolism, lysine biosynthesis, vitamin B6 metabolism, aminoacyl-tRNA biosynthesis, glycolysis or gluconeogenesis, and purine metabolism, were significantly affected. The metabolism of alanine, aspartate, and glutamate was particularly affected [47]. Similarly, significant differences in the asparagine concentration, which is the neutral derivate of aspartate, were observed between the PPIDarr and the nPPID group. Different observations between the mentioned study and our study are probably caused by the different pathogenesis of the diseases.

## 5. Conclusions

Altered amino acid concentrations are found in horses suffering from PPID when compared to healthy horses. Especially asparagine, citrulline, and glutamine may be potential diagnostic markers and may offer interesting approaches for research regarding amino acid supplementation in PPID patients.

## Figures and Tables

**Table 1:** Age and ACTH concentrations of the tested horses (mean ± standard deviation).

Parameter	nPPID	PPID	PPIDrr	PPIDarr
ACTH (pg/mL)	19.76 ± 6.96	382.85 ± 352.69	18.1 ± 7.88	154.0 ± 23.07
Age (years)	15.91 ± 6.96	27.79 ± 6.7	21.25 ± 3.77	27.33 ± 4.16

nPPID: non-PPID horse, PPID: horse suffering from PPID, PPIDrr: horse treated for PPID and ACTH ≤ 30 pg/mL, and PPIDarr: horse treated for PPID and ACTH ≥ 100 pg/mL.

**Table 2:** Mean and standard deviations of the normally distributed amino acid concentrations (nmol/mL); *p*-values (ANOVA).

	nPPID		PPID		PPIDrr		PPIDarr		*p*
	Mean	SD	Mean	SD	Mean	SD	Mean	SD	
1mHis	17.1	6.0	15.8	6.0	18.5	9.2	23.7	6.2	0.231
Ala	205.8	69.0	215.0	85.1	164.2	41.7	213	70.3	0.595
Arg	67.3	29.7	86.7	20.0	95.7	27.1	116.0	16.0	0.004
Asn	35.6	20.3	68.0	29.1	33.0	12.2	78.9	53.1	0.018
Cit	56.4	20.2	53.3	19.1	83.9	12.0	68.1	17.4	0.016
Cys	135.6	81.9	169.8	32.9	18.7	7.4	146.4	115.0	<0.001
GABA	20.4	8.0	19.3	8.0	12.9	1.1	21.9	11.1	0.258
Gln	239.8	56.1	334.8	43.9	286.4	46.0	337.4	42.363	<0.001
Glu	40.9	24.4	35.7	20.3	18.3	5.3	41.4	23.2	0.21
Gly	410.2	161.6	431.9	119.5	400.8	146.7	384.4	90.2	0.92
His	71.2	13.4	81.3	17.2	77.5	8.0	86.4	5.1	0.89
Ile	60.6	16.0	70.2	19.4	53.2	15.4	72.9	11.5	0.113
Leu	100.9	30.6	112.6	36.8	97.8	24.7	123.5	20.7	0.458
Orn	59.5	18.1	57.4	14.3	64.0	5.5	55.7	5.2	0.83
Phe	54.9	11.4	53.5	11.2	60.1	7.2	59.0	3.6	0.603
Ser	212.4	64.6	244.2	68.1	220.9	34.0	227.1	20.5	0.419
Tau	41.3	12.6	47.3	14.6	40.1	13.1	42.9	13.3	0.473
Trp	50.9	12.8	52.2	16.3	56.0	11.5	72.6	12.5	0.102
Val	157.4	52.0	162.7	49.2	172.7	26.5	174.8	42.9	0.88

nPPID: non-PPID horse, PPID: horse suffering from PPID, PPIDrr: horse treated for PPID and ACTH ≤ 30 *p* g/mL, PPIDarr: horse treated for PPID and ACTH ≥ 100 pg/mL, SD: Standard deviation, 1mHis: 1-methyl histidine, Ala: Alanine, Arg: Arginine, Asn: Aspargine; Citr: Citrulline, Cys: Cysteine, GABA: Gamma-aminobutyric acid, Gln: Glutamine, Glu: Glutamic acid, Gly: Glycine, His: Histidine, Ile: Isoleucine, Leu: Leucine, Met: Methionine, Orn: Ornithine, Phe: Phenylalanine, Ser: Serine, Tau: Taurine, Trp: Tryptophan, Val: Valine.

**Table 3:** Median, minimum, and maximum of the non—normally distributed amino acid concentrations (nmol/mL); Kruskal—Wallis Test.

	nPPID			PPID			PPIDrr			PPIDarr			*p*
	Med	Min	Max	Med	Min	Max	Med	Min	Max	Med	Min	Max	
Asp	8.1	5	16.5	5	5	20.7	8.1	5.7	9.9	8.3	5.0	16.5	0.304
Lys	76.1	45.5	153.8	92.6	29.3	143.5	87.8	46.7	122.9	114.6	80.0	126.2	0.498
Pro	76.3	42.8	308.7	74.0	35.8	147.1	68.2	46.6	104.1	102.2	78.2	112.7	0.315
Thr	94.7	41.1	203.9	129.6	65.9	208.4	113.2	39.6	138.6	151.5	125.7	156.6	0.047
Tyr	52.1	36.5	97.6	58.0	36.4	93.5	63.8	46.5	96.7	63.2	50.8	64.4	0.528

nPPID: non-PPID horse, PPID: horse suffering from PPID, PPIDrr: horse treated for PPID and ACTH ≤ 30 *p* g/mL, PPIDarr: horse treated for PPID and ACTH ≥ 100 pg/mL, Med: median, Min: minimum, Max: maximum, Asp: aspartic Acid, Lys: lysine, Pro: proline, Thr: threonine, Tyr: tyrosine.

## Data Availability

Additional data are available from the corresponding author.

## References

[1] Brosnahan M.M., Paradis M.R. (2003). Assessment of clinical characteristics, management practices, and activities of geriatric horses. J. Am. Vet. Med. Assoc..

[2] McGowan T.W., Pinchbeck G., Phillips C.J., Perkins N., Hodgson D.R., McGowan C.M. (2010). A survey of aged horses in Queensland, Australia. Part 2: Clinical signs and owners’ perceptions of health and welfare. Aust. Vet. J..

[3] Ireland J.L., Clegg P.D., McGowan C.M., McKane S.A., Chandler K.J., Pinchbeck G.L. (2012). Disease prevalence in geriatric horses in the United Kingdom: Veterinary clinical assessment of 200 cases. Equine Vet. J..

[4] Van der Kolk J., Kalsbeek H., Van Garderen E., Wensing T., Breukink H. (1993). Equine pituitary neoplasia: A clinical report of 21 cases (1990–1992). Vet. Rec..

[5] Couëtil L., Paradis M.R., Knoll J. (1996). Plasma adrenocorticotropin concentration in healthy horses and in horses with clinical signs of hyperadrenocorticism. J. Vet. Intern. Med..

[6] Hillyer M., Taylor F., Mair T., Murphy D., Watson T., Love S. (1992). Diagnosis of hyperadrenocorticism in the horse. Equine Vet. Educ..

[7] Hart K.A., Goff J.P., McFarlane D., Breuhaus B., Frank N., De Laat M.A., McGowan C.M., Toribio R.E., Bauman D.E., Collier R.J., Smith B.P., van Metre D.C., Pusterla N. (2019). Endocrine and Metabolic Disease. Large Animal Internal Medicine.

[8] Orth D.N., Holscher M.A., Wilson M.G., Nicholson W.E., Plue R.E., Mount C.D. (1982). Equine Cushing’s disease: Plasma immunoreactive proopiolipomelanocortin peptide and cortisol levels basally and in response to diagnostic tests. Endocrinology.

[9] Horowitz M., Neal L., Watson J. (2003). Characteristics of plasma adrenocorticotropin, β-endorphin and α-melanocyte stimulating hormone as diagnostic tests for pituitary pars intermedia dysfunction in the horse. J. Vet. Intern. Med..

[10] McFarlane D., Breshears M.A., Cordero M., Hill K., Carmichael R., Maxwell L.K. (2012). Comparison of plasma ACTH concentration, plasma α-MSH concentration, and overnight dexamethasone suppression test for diagnosis of PPID. Proc. ACVIM.

[11] Arlt W., Stewart P.M. (2005). Adrenal corticosteroid biosynthesis, metabolism, and action. Endocrinol. Metab. Clin..

[12] Tiley H.A., Geor R.J., McCutcheon L.J. (2007). Effects of dexamethasone on glucose dynamics and insulin sensitivity in healthy horses. Am. J. Vet. Res..

[13] Haffner J., Eiler H., Hoffman R., Fecteau K., Oliver J. (2009). Effect of a single dose of dexamethasone on glucose homeostasis in healthy horses by using the combined intravenous glucose and insulin test. J. Anim. Sci..

[14] Firshman A.M., Valberg S.J., Karges T.L., Benedict L.E., Annandale E.J., Seaquist E.R. (2005). Serum creatine kinase response to exercise during dexamethasone-induced insulin resistance in Quarter Horses with polysaccharide storage myopathy. Am. J. Vet. Res..

[15] Cartmill J., Thompson D., Storer W., Crowley J., Huff N., Waller C. (2005). Effect of dexamethasone, feeding time, and insulin infusion on leptin concentrations in stallions. J. Anim. Sci..

[16] Bailey S.R., Menzies-Gow N.J., Harris P.A., Habershon-Butcher J.L., Crawford C., Berhane Y., Boston R.C., Elliott J. (2007). Effect of dietary fructans and dexamethasone administration on the insulin response of ponies predisposed to laminitis. J. Am. Vet. Med. Assoc..

[17] Tiley H.A., Geor R.J., McCutcheon L.J. (2008). Effects of dexamethasone administration on insulin resistance and components of insulin signaling and glucose metabolism in equine skeletal muscle. Am. J. Vet. Res..

[18] Sandow C., Fugler L.A., Leise B., Riggs L., Monroe W.T., Totaro N., Belknap J., Eades S. (2019). Ex vivo effects of insulin on the structural integrity of equine digital lamellae. Equine Vet. J..

[19] de Laat M.A., Pollitt C.C., Kyaw-Tanner M.T., McGowan C.M., Sillence M.N. (2013). A potential role for lamellar insulin-like growth factor-1 receptor in the pathogenesis of hyperinsulinaemic laminitis. Vet. J. (Lond. Engl. 1997).

[20] Lane H.E., Burns T.A., Hegedus O.C., Watts M.R., Weber P.S., Woltman K.A., Geor R.J., McCutcheon L.J., Eades S.C., Mathes L.E. (2017). Lamellar events related to insulin-like growth factor-1 receptor signalling in two models relevant to endocrinopathic laminitis. Equine Vet. J..

[21] Stokes S.M., Stefanovski D., Bertin F.-R., Medina-Torres C.E., Belknap J.K., van Eps A.W. (2021). Plasma amino acid concentrations during experimental hyperinsulinemia in 2 laminitis models. J. Vet. Intern. Med..

[22] Fukagawa N.K., Minaker K.L., Young V.R., Rowe J.W. (1986). Insulin dose-dependent reductions in plasma amino acids in man. Am. J. Physiol..

[23] Urschel K.L., Escobar J., McCutcheon L.J., Geor R.J. (2014). Insulin infusion stimulates whole-body protein synthesis and activates the upstream and downstream effectors of mechanistic target of rapamycin signaling in the gluteus medius muscle of mature horses. Domest. Anim. Endocrinol..

[24] Hillier T.A., Fryburg D.A., Jahn L.A., Barrett E.J. (1998). Extreme hyperinsulinemia unmasks insulin’s effect to stimulate protein synthesis in the human forearm. Am. J. Physiol..

[25] Timmerman K.L., Lee J.L., Dreyer H.C., Dhanani S., Glynn E.L., Fry C.S., Drummond M.J., Sheffield-Moore M., Rasmussen B.B., Volpi E. (2010). Insulin stimulates human skeletal muscle protein synthesis via an indirect mechanism involving endothelial-dependent vasodilation and mammalian target of rapamycin complex 1 signaling. J. Clin. Endocrinol. Metab..

[26] Tuvdendorj D., Børsheim E., Sharp C.P., Zhang X., Barone C.M., Chinkes D.L., Wolfe R.R. (2015). Amino Acid Availability Regulates the Effect of Hyperinsulinemia on Skin Protein Metabolism in Pigs. J. Biol. Chem..

[27] McFarlane D. (2007). Advantages and limitations of the equine disease, pituitary pars intermedia dysfunction as a model of spontaneous dopaminergic neurodegenerative disease. Ageing Res. Rev..

[28] Figura M., Kuśmierska K., Bucior E., Szlufik S., Koziorowski D., Jamrozik Z., Janik P. (2018). Serum amino acid profile in patients with Parkinson’s disease. PLoS ONE.

[29] Naushad S.M., Jain J.M.N., Prasad C.K., Naik U., Akella R.R.D. (2013). Autistic children exhibit distinct plasma amino acid profile. NISCAIR-CSIR.

[30] Bala K., Dogan M., Mutluer T., Kaba S., Aslan O., Balahoroglu R., Cokluk E., Ustyol L., Kocaman S. (2016). Plasma amino acid profile in autism spectrum disorder (ASD). Eur. Rev. Med. Pharm. Sci..

[31] Rees C.A., Rostad C.A., Mantus G., Anderson E.J., Chahroudi A., Jaggi P., Wrammert J., Ochoa J.B., Ochoa A., Basu R.K. (2021). Altered amino acid profile in patients with SARS-CoV-2 infection. Proc. Natl. Acad. Sci. USA.

[32] Lai H.-S., Lee J.-C., Lee P.-H., Wang S.-T., Chen W.-J. (2005). Plasma free amino acid profile in cancer patients. Semin. Cancer Biol..

[33] Proenza A.M., Oliver J., Palou A., Roca P. (2003). Breast and lung cancer are associated with a decrease in blood cell amino acid content. J. Nutr. Biochem..

[34] Cangiano C., Muscaritoli M., Conversano L., Torelli G., Cascino A. (1995). Tumor-induced changes in host metabolism: A possible marker of neoplastic disease. Nutrition (Burbank Los Angeles Cty. Calif.).

[35] Kawamura I., Moldawer L.L., Keenan R.A., Batist G., Bothe A., Bistrian B.R., Blackburn G.L. (1982). Altered amino acid kinetics in rats with progressive tumor growth. Cancer Res..

[36] Pisters P.W., Pearlstone D.B., Toroslan M. (1993). Protein and amino acid metabolism in cancer cachexia: Investigative techniques and therapeutic interventions. Crit. Rev. Clin. Lab. Sci..

[37] Heslin M.J., Newman E., Wolf R.F., Pisters P.W., Brennan M.F. (1992). Effect of systemic hyperinsulinemia in cancer patients. Cancer Res..

[38] Kenéz Á., Warnken T., Feige K., Huber K. (2018). Lower plasma trans-4-hydroxyproline and methionine sulfoxide levels are associated with insulin dysregulation in horses. BMC Vet. Res..

[39] Delarocque J., Frers F., Feige K., Huber K., Jung K., Warnken T. (2021). Metabolic changes induced by oral glucose tests in horses and their diagnostic use. J. Vet. Intern. Med..

[40] Chandler K.J., Mellor D.J. A pilot study of the prevalence of disease within a geriatric horse population. Proceedings of the 40th Congress of the British Equine Veterinary Association.

[41] McGowan T.W., Pinchbeck G.P., McGowan C.M. (2013). Prevalence, risk factors and clinical signs predictive for equine pituitary pars intermedia dysfunction in aged horses. Equine Vet. J..

[42] Ireland J.L., Clegg P.D., McGowan C.M., McKane S.A., Pinchbeck G.L. (2011). A cross-sectional study of geriatric horses in the United Kingdom. Part 2: Health care and disease. Equine Vet. J..

[43] Ireland J.L., McGowan C.M., Clegg P.D., Chandler K.J., Pinchbeck G.L. (2012). A survey of health care and disease in geriatric horses aged 30 years or older. Vet. J..

[44] Paradis M.R. (2002). Demographics of health and disease in the geriatric horse. Vet. Clin. N. Am. Equine Pract..

[45] Frank N., Andrews F.M., Sommardahl C.S., Eiler H., Rohrbach B.W., Donnell R.L. (2006). Evaluation of the combined dexamethasone suppression/ thyrotropin-releasing hormone stimulation test for detection of pars intermedia pituitary adenomas in horses. J. Vet. Intern. Med..

[46] Hart K., Durham A., Frank N., McGowan C., Schott H., Stewart A. (2021). The Equine Endocrinology Group (EEG) Pituitary Pars intermedia (PPID). https://sites.tufts.edu/equineendogroup/files/2021/12/2021-PPID-Recommendations-V11-wo-insert.pdf.

[47] Oklu R., Deipolyi A.R., Wicky S., Ergul E., Deik A.A., Chen J.W., Hirsch J.A., Wojtkiewicz G.R., Clish C.B. (2014). Identification of small compound biomarkers of pituitary adenoma: A bilateral inferior petrosal sinus sampling study. J. Neurointerv. Surg..

[48] De Vries J.L. (2015). Mechanism of Intestinal and Skeletal Muscle Glucose and Amino Acid Uptake in Pituitary Pars Intermedia Dysfunction.

[49] Krebs H.A. (1935). Metabolism of amino-acids: The synthesis of glutamine from glutamic acid and ammonia, and the enzymic hydrolysis of glutamine in animal tissues. Biochem. J..

[50] MacLennan P.A., Smith K., Weryk B., Watt P.W., Rennie M.J. (1988). Inhibition of protein breakdown by glutamine in perfused rat skeletal muscle. FEBS Lett..

[51] MacLennan P.A., Brown R., Rennie M.J. (1987). A positive relationship between protein synthetic rate and intracellular glutamine concentration in perfused rat skeletal muscle. FEBS Lett..

[52] Souba W.W. (1991). Glutamine: A key substrate for the splanchnic bed. Annu. Rev. Nutr..

[53] Lacey J.M., Wilmore D.W. (1990). Is glutamine a conditionally essential amino acid?. Nutr. Rev..

[54] Crenn P., Messing B., Cynober L. (2008). Citrulline as a biomarker of intestinal failure due to enterocyte mass reduction. Clin. Nutr..

[55] Crenn P., Cynober L. (2010). Effect of intestinal resections on arginine metabolism: Practical implications for nutrition support. Curr. Opin. Clin. Nutr. Metab. Care.

[56] Curis E., Nicolis I., Moinard C., Osowska S., Zerrouk N., Bénazeth S., Cynober L. (2005). Almost all about citrulline in mammals. Amino Acids.

[57] Moinard C., Cynober L. (2007). Citrulline: A new player in the control of nitrogen homeostasis. J. Nutr..

[58] Dias R.G., Negrão C.E., Krieger M.H. (2011). Nitric oxide and the cardiovascular system: Cell activation, vascular reactivity and genetic variant. Arq. Bras. Cardiol..

[59] Tousoulis D., Kampoli A.-M., Tentolouris Nikolaos Papageorgiou C., Stefanadis C. (2012). The role of nitric oxide on endothelial function. Curr. Vasc. Pharmacol..

[60] Ardigo D., Stüehlinger M., Franzini L., Valtuena S., Piatti P., Pachinger O., Reaven G., Zavaroni I. (2007). ADMA is independently related to flow-mediated vasodilation in subjects at low cardiovascular risk. Eur. J. Clin. Investig..

[61] Lau T., Owen W., Yu Y.M., Noviski N., Lyons J., Zurakowski D., Tsay R., Ajami A., Young V.R., Castillo L. (2000). Arginine, citrulline, and nitric oxide metabolism in end-stage renal disease patients. J. Clin. Investig..

[62] Wu G. (1998). Intestinal mucosal amino acid catabolism. J. Nutr..

[63] Romero M.J., Platt D.H., Caldwell R.B., Caldwell R.W. (2006). Therapeutic use of citrulline in cardiovascular disease. Cardiovasc. Drug Rev..

[64] Jackson A.L. (2013). Plasma Citrulline Levels in Horses at Risk of Acute Laminitis. Ph.D. Dissertation.

[65] Karikoski N.P., Horn I., McGowan T.W., McGowan C.M. (2011). The prevalence of endocrinopathic laminitis among horses presented for laminitis at a first-opinion/referral equine hospital. Domest. Anim. Endocrinol..

[66] Marino S.M., Gladyshev V.N. (2010). Cysteine Function Governs Its Conservation and Degeneration and Restricts Its Utilization on Protein Surfaces. J. Mol. Biol..

[67] Wu H., Ma B.-G., Zhao J.-T., Zhang H.-Y. (2007). How similar are amino acid mutations in human genetic diseases and evolution. Biochem. Biophys. Res. Commun..

[68] Fomenko D.E., Xing W., Adair B.M., Thomas D.J., Gladyshev V.N. (2007). High-throughput identification of catalytic redox-active cysteine residues. Science.

[69] Sei C., Toneff T., Aaron W., Hook V.Y.H. (2003). Regulation of ACTH levels in anterior pituitary cells during stimulated secretion: Evidence for aspartyl and cysteine proteases in the cellular metabolism of ACTH. Peptides.

